# Using deliberate practice framework to assess the quality of feedback in undergraduate clinical skills training

**DOI:** 10.1186/s12909-019-1547-5

**Published:** 2019-04-11

**Authors:** Reina M. Abraham, Veena S. Singaram

**Affiliations:** Clinical and Professional Practice, School of Clinical Medicine, College of Health Sciences, University of KwaZulu-Natal, Durban, 4000 South Africa

**Keywords:** Medical education, Feedback, Evaluation, Deliberate practice, Feed-forward, Clinical skills

## Abstract

**Background:**

In this research paper we report on the quality of feedback provided in the logbooks of pre-clinical undergraduate students based on a model of ‘actionable feedback’. Feedback to clinical learners about their performance is crucial to their learning, which ultimately impacts on their development into competent clinicians. Due to students’ concerns regarding the inconsistency and quality of feedback provided by clinicians, a structured feedback improvement strategy to move feedback forward was added to the clinical skills logbook. The instrument was also extended for peer assessment. This study aims to assess the quality of feedback using the deliberate practice framework.

**Methods:**

A feedback scoring system was used to retrospectively assess the quality of tutor and peer logbook feedback provided to second and third year medical students to identify deliberate practice components i.e. task, performance gap and action plan. The sample consisted of 425 second year and 600 third year feedback responses over a year.

**Results:**

All three deliberate practice components were observed in the majority of the written feedback for both classes. The frequency was higher in peer (83%, 89%) than tutor logbook assessments (51%, 67%) in both classes respectively. Average tutor and peer task, gap and action feedback scores ranged from 1.84–2.07 and 1.93–2.21 respectively. The overall quality of feedback provided by the tutor and peer was moderate and less specific (average score < or = 2). The absence of the three components was noted in only 1% of the feedback responses in both 2nd and 3rd year.

**Conclusion:**

This study found that adding in a feed-forward strategy to the logbooks increased the overall quality of tutor and peer feedback as the task, gap and action plans were described. Deliberate practice framework provides an objective assessment of tutor and peer feedback quality and can be used for faculty development and training. The findings from our study suggest that the ratings from the tool can also be used as guidelines to provide feedback providers with feedback on the quality of feedback they provided. This includes specifically describing a task, performance gap and providing a learning plan as feed-forward to enhance feedback given.

**Electronic supplementary material:**

The online version of this article (10.1186/s12909-019-1547-5) contains supplementary material, which is available to authorized users.

## Background

Medical students view feedback as a valuable component for improving their learning [1, 2]. In medical education feedback is defined as “specific information about the comparison between trainees’ observed performance and a standard, given with the intent to improve the trainee’s performance” [3]. Without feedback good performances are not supported and mistakes remain [4]. How feedback translates into improved clinical performance is poorly studied [5]. There is the need to understand the mechanism by which feedback leads to improved performance [3]. A good assessment not just evaluates whether competencies are defined alongside the related learning, it likewise creates new learning and is oriented towards improvement. There is a need for change from an assessment “of” learning to an assessment “for” learning [6]. Apart from developing different assessment tasks to accomplish this shift, there is likewise a need to change the manner in which students are informed about the learning evaluation results (feedback) and how to make decisions from these results (feed-forward) [5, 6, 26]. Studies have described both feedback process [7–10] and content [3, 10, 11] as important factors for improved clinical performance. The use of these factors to assess the quality of feedback is less common [5].

Student doctor’s clinical skills development is affected by many factors making it difficult to directly study the impact of feedback on clinical performance. If expertise development is the goal of formative assessment then using Ericsson’s model of deliberate practice to evaluate feedback quality would be useful [12]. Ericsson introduced the concept of ‘deliberate practice’ characterizing training as “highly structured activities explicitly directed at improvement of performance in a particular domain” with the aim of reaching a well-defined goal to improve skills performance [12]. Deliberate practice, a way of competency-based skills development includes baseline assessment of clinical performance, immediate specific directly observed feedback, opportunities to improve through repetition and subsequent measurement of clinical performance [13–15]. Deliberate practice with clear specific tasks and feedback following oral presentations [16] and objective structured clinical examination (OSCE) [17] has had a positive effect on the acquisition of skills and improved clinical performance.

Feedback quality was often evaluated in medical education as confirmative or corrective based on the presence or absence of features of an effective feedback [18, 19]. To promote learning, effective feedback processes should also contain elements that facilitate deliberate practice to help learners understand their subject area and give them clear guidelines on how to improve their learning. The belief that feedback can be used by students to enhance their learning and inform their efforts in future assessments encapsulates the notion of ‘feed-forward’. Learners therefore need to know the task related learning goals, their performances directly observed and compared with this standard to inform them of their learning needs and knowledge gaps. Prompt action to motivate learners to drive learning forward by reducing this performance gap is also necessary [1, 20].

Despite educators striving to provide high quality feedback, students frequently report poor quality feedback [20–22]. Providing continuous effective feedback from different sources such as tutors and peers can also increase the impact of the logbook as a formative assessment tool and feedback instrument to guide learning, reduce the assessment gap and increase reflection and reliability [23–25]. It is important for feedback to contain specific comments that facilitate reflection and action plans [26]. Early simulation of deliberate practice in a simulated setting such as the clinical skills laboratory also enhances competency-based skills development and transference of skills to the clinical setting [15, 26].

As described in the literature, logbooks are used globally to “provide a clear setting of learning objectives and give trainees and clinical teachers a quick overview of the requirements of training and an idea of the learning progress” [27]. However, in a previous study on student’s perceptions of logbook feedback in our clinical skills setting, comments were found to be vague and inconsistent [22]. To address this, a structured feedback improvement strategy providing a forward direction was added to the logbook [22]. Using Ericsson’s theory of deliberate practice, a key component of expertise development, this study aims to evaluate the quality of written feedback provided to pre-clinical undergraduate medical students in the clinical skills laboratory during formative logbook assessments following the feedback improvement intervention. A modified and adapted feedback-scoring tool based on a deliberate practice framework [5] was used to investigate and provide answers to the following: Can components that facilitate deliberate practice be identified in the feedback provided to medical learners? To what extent does the feedback provided contain elements that facilitate deliberate practice? Is there a difference in the quality of feedback provided by the tutors and peers?

## Methods

### Context and setting

This study was carried out at the Nelson R Mandela School of Medicine (NRMSM), University of KwaZulu-Natal (UKZN) clinical skills laboratory. The role of the clinical tutors during the clinical skills sessions follows the same teaching stages as proposed by Barr: The tutor first demonstrates the skill while the student’s observes [28]. The tutor then discusses the outcomes of the skill with the students. The students demonstrate the skill while the tutor observes and coaches the students. The students then receive feedback on their clinical performance from the tutor and finally the student is left to work independently once they have mastered the necessary clinical skills. At the end of a six-week theme students are assessed formatively and provided with immediate directly observed verbal and written feedback in their logbooks for later reference along with a global rating of superior performance, competent or failure by supervising clinical tutors and peers. Students are informed that a mark will not be given being a formative assessment but the rating will assist in understanding their level of skill mastery. To enhance the logbook feedback a feed-forward strategy on what was done well, what was not done well and what can be improved was incorporated into the logbook which allowed clinicians and peers to provide students with learning goals/action plans targeting the performance assessment process and not just the assessment product. These changes to enhance constructive feedback were communicated to both the tutors and students via formal information sessions. All clinical skills protocols are included in the logbooks to ensure that students are familiar with the expected performance standards. Students are often supervised and assessed by more than one clinical tutor and peer and each clinical tutor and peer assesses more than one student during each theme.

### Study design

#### Study population, sample size and sampling method

This retrospective cross-sectional study analysed the logbooks from twenty five 2nd and thirty 3rd year students that were randomly selected from each category of high achievers (HA) (> 70%), average achievers (AA) (50–69%) and low achievers (LA) (< 50%) based on their end of year summative OSCE assessment performance. A maximum variation sampling approach ensured the sample included logbooks of students with a wide ranch of achievement in clinical skills and who had at least one year of exposure to the clinical skills logbook formative assessment feedback. Logbook feedback forms (Additional files 1 and 2) for each student category completed over a year were included in the study. A total of 425 second year and 600 third year entries were included in the study sample.

#### Data collection and adaptation of the scoring tool

The logbook feedback was analysed using a tool designed by Gauthier et al. based on the deliberate practice framework to determine for the presence and extent of the three components that facilitate deliberate practice [5]. This tool was adapted and modified to our learning environment (Table 1) and used to assess all feedback responses for the presence of deliberate practice components as outlined in Table 1: (1) Task: What was done well with regards to a well-defined goal/task, (2) Gap: What was not done well and identification of a gap between observed current performance and a standard, (3) Action: What can be improved and if a learning or action plan was provided. Each component was scored from 0 to 3 (0 = absent, 1 = alluded to the component or vaguely described, 2 = general mention of the component, 3 = specific mention of the component) to ensure components could be objectively separated by specificity to warrant rater reliability and to differentiate a good from a poor quality feedback [5].

**Table 1:** Task, gap and action feedback scoring table adapted from Gauthier et al. (2015)

	0	1	2	3
Task – What was done well? (A description of the event around which feedback was given)	Task not Described	*Vague*. Lacking either content or value. (No specific behaviour was identified with regards to the learning goal for the task e.g. ‘You did great’)	Content or value described *generally* (A general description of the behaviour was identified with regards to the learning goal for the task e.g. ‘General examination done, Inspection of the chest done, auscultation done’)	*Specific*. Content or value specifically described. (A good description of the steps to the particular task/skill provided e.g. Positioned the patient correctly to examine the chest, when examining for aortic regurgitation had the patient lean forward and exhale)
Gap – What was not done well? (The recognition of a difference between their performance and that of a comparative standard)	No gap Described	Gap *alluded to*. (No suggestions geared toward identified behaviour. e.g. ‘Your technique was awful’)	Gap *generally* described. (Concise issue raised but limited suggestions provided to learner e.g. You looked very uncomfortable examining that chest’)	*Specific* gap identified. (Concise issues identified and learner provided with information to close a gap in knowledge e.g. ‘Your exam of the chest was appropriate but percussion technique was inadequate. You may be more comfortable if you position your fingers on the chest this way’)
Action – What can be improved? (Using the feedback to create a future learning goal or plan)	No learning goal or plan.	Learning goal or plan *alluded to*. (Feedback terminated with no plans for follow-up or re-evaluation e.g. ‘Great job’)	*General* goal or plan described. (Broad action plan is suggested but not specific to behaviour or encounter e.g. ‘Read more around your cases’)	*Specific* goal or plan described. (Clear plan to modify or reinforce behaviour e.g. ‘Read this article on chest examination, practice the percussion technique and I will watch you examine the next patient with pneumonia’)

Two clinician raters independently assessed all written feedback included in the study for the presence of the three components of deliberate practice. The raters included the researcher and one clinician in the faculty with direct involvement in educational activities in the clinical skills laboratory. The raters initially familiarised themselves with the original feedback scoring tool developed by Gauthier et al. [5]. To increase reliability raters independently scored a small selection of the same logbook written feedback responses followed by comparing scores and discussions about difficulties and discrepancies with the descriptions of each scoring element. To enhance the discrimination between scores, specific behavioural anchors for each scoring item was added to the individual descriptions of the deliberate practice elements to adapt the scoring tool to our clinical skills environment (Table 1) as this has been shown to increase clarity [29] and inter-rater reliability [30]. The feedback responses were then scored separately using the modified task, gap and action grading tool. Inter-rater reliability was analysed by averaging discrepancies between scores and the Cohen’s kappa coefficient (k) calculated to measure inter-rater agreement [31].

#### Data analysis

Written comments that was evaluated using the adapted scoring system [5] identified and discriminated a low quality feedback (score 0–1) from a moderate quality (score of 2) and a more specific high quality feedback (score of 3). The primary outcome measures for our study included the frequency distribution (i.e. the number of comments in each feedback category of task, gap and action (TGA) was counted and aggregated on a percentage (frequency) basis) and average scores of TGA as indicated in the written feedback of all logbook skills encounters assessed in the three categories (HA, AA and LA) of 2nd (17 skills/student) and 3rd year (20 skills/student) medical students.

A Z-test for difference of two proportions was conducted separately on each of the variables (task, gap and action) as well as variations with year of study and feedback source. The Kolmogorov Smirnov test was used to assess the normality of feedback scores. The Kruskal Wallis test was then used to compare the average deliberate practice component scores based on academic performance for the three categories of students (HA, AA and LA). Proportions between the global ratings and component scores (TGA) was investigated using the Fischer’s exact test. A *p* value less than 0.05 were deemed statistically significant. All statistical analyses were performed using SPSS version 25.

## Results

One thousand and twenty five written feedback responses from 55 logbooks were assessed. Table 2 represents characteristics of the feedback entries. Eight evaluations in the 2nd year category and 35 evaluations in the 3rd year category were left blank as the students did not attempt these skills.

**Table 2:** Characteristics of the 2nd and 3rd year clinical skills logbook encounters

Time period Jan 2017 – Dec 2017	2nd year evaluations	3rd year evaluations
Feedback entries, N:	425	600
Participant/Evaluator characteristics:		
Number of students/logbooks	25	30
Number of tasks assessed per student (range)	1–17	1–20
Number of clinical tutors	10	10
Number of tasks assessed per clinical tutor (range)	1–12	1–10
Number of peers (range)	50–100	50–100
Number of tasks assessed per peer (range)	1–30	1–30
Encounter focus:		
Physical examination skills (2 tutor and 4 peer assessed)	7 (40%)	6 (30%)
Procedural skills (all peer assessed)	10 (60%)	14 (70%)
Category of students assessed based on end of year OSCE marks:		
Low achievers (< 50%)	5 (20%)	10 (38%)
Average achievers (50–69%)	10 (40%)	10 (30%)
High achievers (> 70%)	10 (40%)	10 (31%)

The kappa correlation coefficient obtained between ratings assigned by the two raters were all high (r > 0.8 for all comparisons) with no significant differences between raters suggesting a near perfect agreement with both raters producing similar scores to the same data item while using the feedback scoring Table.

### A. Assessment of proportion of deliberate practice elements identified in the written feedback comments

**Fig. 1:**
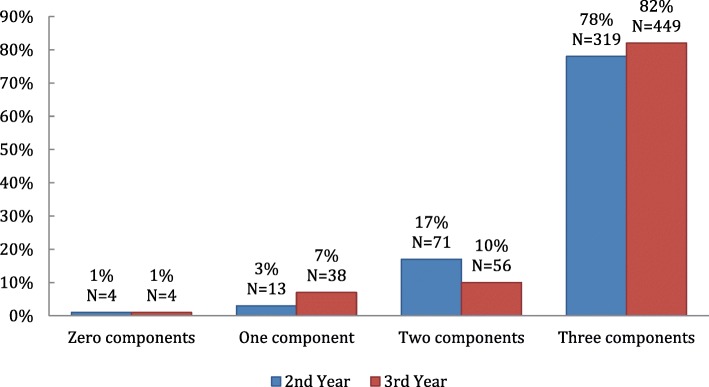
Proportion of components of deliberate practice identified in all written feedback comments in 2nd and 3rd year logbooks

**Fig. 2:**
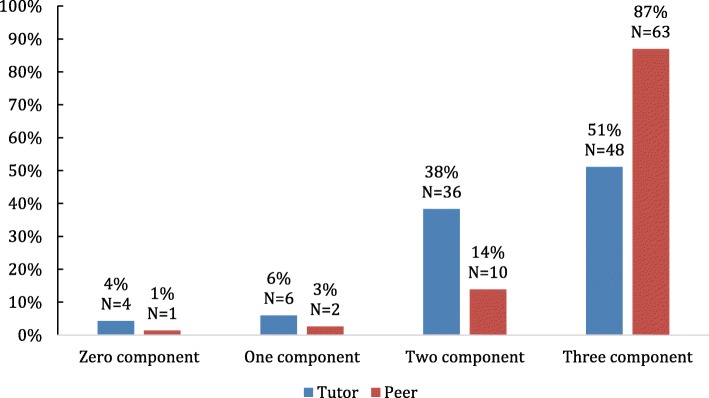
Proportion of components of deliberate practice identified in tutor and peer written feedback comments in the 2nd year logbooks

We measured the frequency with which none, one, two or all three components of deliberate practice (TGA) were identified in the feedback. The frequency with which it was possible to identify these components in the written feedback evaluation is represented in Figs. 1 and 2.

#### All feedback – 2nd and 3rd year

In this study we found that all three components of deliberate practice were identified in 78% of the 2nd and 82% of the 3rd year logbooks (Fig. 1). The absence of three components was noted in only 1% of the feedback responses in both 2nd and 3rd year.

#### Tutors and peer feedback

All three components of deliberate practice were identified in 51% of the tutor and 87% of peer feedback responses in 2nd year logbooks. Similarly 67% of tutor and 89% of peer feedback contained all three components of deliberate practice in the 3rd year logbooks. The absence of the three components were noted in only 4% and 1% of the tutor and peer feedback respectively (Fig. 2).

### B. Assessment of the degree of each component of deliberate practice identified in the written feedback comments

**Fig. 3:**
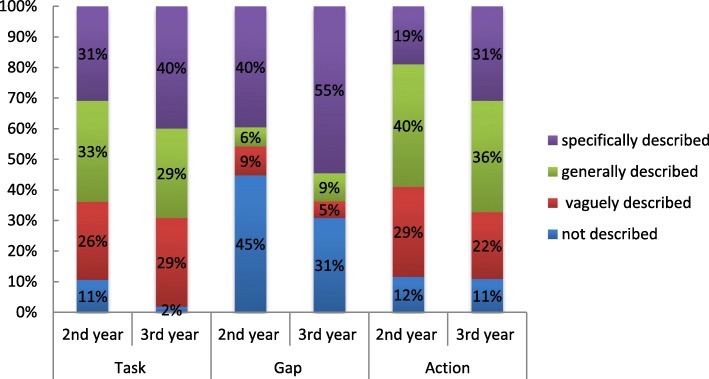
Assessment of degree of each component of deliberate practice in 2nd and 3rd year tutor feedback

**Fig. 4:**
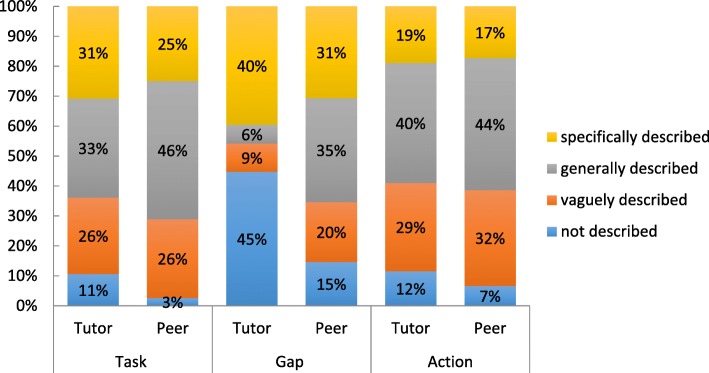
Assessment of degree of each component of deliberate practice in 2nd year tutor and peer feedback

We assessed the degree of each component of deliberate practice (TGA) in the feedback comments as follows: 0–3 (0 = not described, 1 = vaguely described, 2 = generally described, 3 = specifically described). The results are illustrated in Figs. 3 and 4.

#### Tutor feedback – 2nd and 3rd year

Figure 3 summarizes the degree of deliberate practice components in tutor feedback in the 2nd and 3rd year logbooks. The tutor feedback on the task, gap and action to the 3rd year students were more specifically described compared to the 2nd year students.

Specific task (40%, 31%), gap (55%, 40%) and action (31%, 19%) were identified more often in the 3rd year feedback compared to the 2nd year feedback comments respectively. General task (33%, 29%) and action (40%, 36%) were identified more frequently in 2nd year compared to the 3rd year feedback respectively. No gap (45%, 31%) was identified more often in the 2nd year compared to the 3rd year feedback responses respectively. The difference of proportions between the deliberate practice task, gap and action feedback scores for each skill assessed was statistically significant (*p* < 0.05) between the 2nd and 3rd year feedback responses. A significant decrease in the specific description of task, gap and action in the 2nd year feedback was found when compared to the 3rd year feedback responses.

#### Tutor and peer feedback

Specific task, gap and action were identified more often in the tutor than the peer feedback as illustrated in Fig. 4.

Specific task (31%, 25%), gap (40%, 31%) and action (19%, 17%) were identified more often in tutor compared to peer feedback respectively. General task (46%, 33%) and action (44%, 40%) were identified more frequently in peer comments compared to the tutor comments respectively. No gap (45%, 15%) was identified more often in tutor feedback compared to peer feedback respectively. When comparing the tutor and peer feedback responses the difference of proportions between the deliberate practice task, gap and action feedback scores for each skill assessed was statistically significant (*p* value < 0.05) indicating a significant decrease in the specific description of task, gap and action in the peer feedback compared to the tutor feedback responses.

### C. Assessment of average deliberate practice component scores based on academic performance

**Fig. 5:**
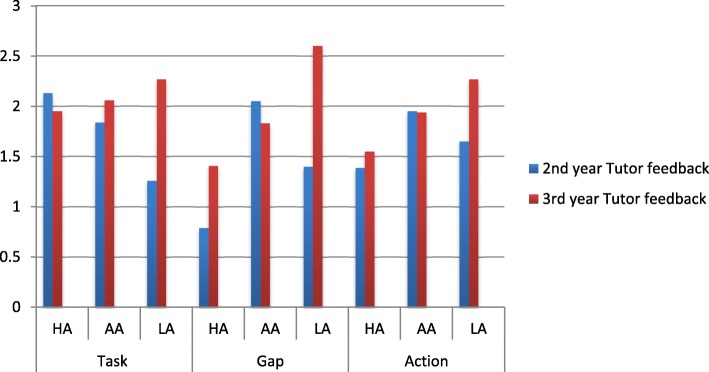
Assessment of average deliberate practice component scores in tutor feedback for the three categories of 2nd and 3rd year students [HA (> 70%); AA (50–69%); LA (< 50%)]

**Fig. 6:**
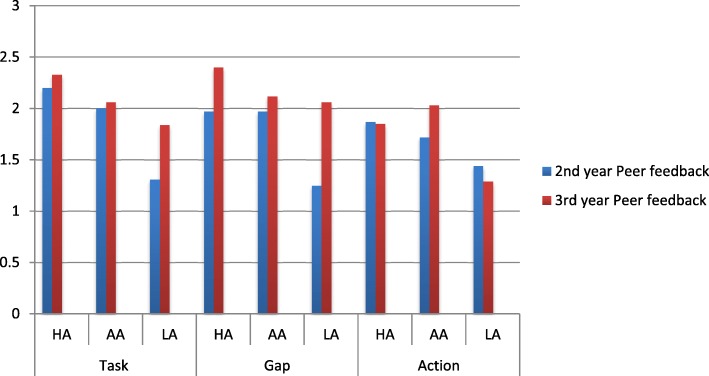
Assessment of average deliberate practice component scores in peer feedback for the three categories of 2nd and 3rd year students [HA (> 70%); AA (50–69%); LA (< 50%)]

We assessed the average deliberate practice component scores in the feedback for the three categories of students (HA, AA and LA) based on their level of achievement and summative marks. The results are illustrated in Figs. 5 and 6.

#### Tutor feedback – 2nd and 3rd year

Average component scores for skills assessed by the tutors plotted for the three different assessment categories of 2nd and 3rd year students is shown in Fig. 5. Overall a statistically significant inverse trend was found when comparing the 3rd year student achievement category with the average task gap and action feedback scores – the higher the student marks the lower was the task, gap and action feedback scores (*p* < 0.05).

The average component scores for tutor feedback on the task, gap and action provided to the LA in the 3rd year were higher than in the 2nd year. The overall quality of the feedback provided by the tutors to the 3rd year was better than that provided to the 2nd year students. The overall quality of feedback provided by the tutors was moderate and less specific (average score < or = 2).

#### Peer feedback – 2nd and 3rd year

The average 2nd and 3rd year peer feedback component scores for the three categories of students are illustrated in Fig. 6. Overall an opposite trend to the tutors is found when comparing the student achievement category with the average task gap and action scores – as the level of achievement increases the task gap and action scores increases (*p* < 0.05).

Similarly the average deliberate practice component scores for peer feedback on the task and gap provided to the HA, AA and LA in the 3rd year were higher than in the 2nd year. The overall quality of the feedback provided by the peers to the 3rd year was better than that provided to the 2nd year students. The overall quality of feedback provided by the peers was moderate and less specific (average score < or = 2).

##### Global rating

An association between the global rating of the students clinical skills development as ‘failure’, ‘competent’ and ‘superior performance’ provided by the tutors and peers and each of the components of deliberate practice was assessed statistically using the Fischer’s test. All the 2nd and 3rd year students were rated as either ‘superior performance’ or ‘competent’. No student was rated ‘failure’. The association between global rating for each skill and the deliberate practice task, gap and action feedback score was statistically significant with a *p* value < 0.05 indicating a statistically significant decrease in gap and action scores as global ratings increased.

## Discussion

High quality feedback motivates learners, is corrective as it confirms learners are on the right path and promotes self-reflection [1]. Since feedback has been shown to be of variable quality and effectiveness, an objective assessment of feedback quality identifies competence in feedback provision and features of highly effective feedback [32]. This study found that majority of the tutor and peer written logbook feedback provided to the medical students contained all three components likely to facilitate deliberate practice, suggesting an implicit adoption of a deliberate practice framework. They were however found more often in the peer than the tutor feedback. Our findings are similar to a previous study by Falchikov who reported that peers provided more positive feedback as well as more prompts and suggestions for improvement than tutors [33]. Nicol indicated that peers tackling the same skill might be able to write more meaningful and relevant comments in a student-centred discourse and get to see examples of good and poor work produced by other students [34]. Engaging students to comment on the work of their peers has the advantage of enhancing their ability to evaluate their own work and improve their self-regulatory skills.

In order for feedback to be effective and of good quality it should be specific [3, 11, 35]. Analysis of both the tutor and peer feedback quality in this study found the performance gap component most often specifically described while the task and action component generally described. Tutors and peers should aim to provide ‘perfectly accurate’ feedback as described by Ericsson with clearly described gaps in knowledge and general strategies for improvement in order for students to undertake sustained ‘deliberate practice’ to progress towards expertise [13]. However it is important to note that developing a learning and action plan should be the responsibility of the feedback receiver. The feedback provider may only facilitate this process as providing too much feedback may inhibit self-directed learning.

The overall quality of the tutor feedback provided to the 3rd year students was better than that provided to the 2nd year students. This finding may be influenced by the student-teacher relationship that plays an important role in the delivery and acceptance of feedback. As the time spent between the two increases, the students mature and become more open minded and accepting of the teaching methods and feedback supplied by the teachers. Additionally, with greater time spent, the teachers begin to understand students and adapt their delivery of feedback in a manner that the student receives and accepts the said feedback better. Bok et al. showed that when medical students build a relationship over time with their clinical tutors there is alignment of the tutor’s goals with their own and they trust the credibility of the feedback they receive [36]. A study exploring medical student’s perceptions of assessment and feedback in a longitudinal integrated clerkship found that a trusting teacher-learner relationship that develops allows “constructive interpretation of critical feedback” with students often supportive of even challenging or corrective feedback [37] making it easier for teachers to provide corrective feedback. The concept of the ‘educational alliance’ framework further recognises the centrality of teacher-learner relationship in the feedback process and its link to the impact of feedback generated within it [38].

In our study, there were certain factors associated with variation in the identification of feedback components and hence the quality of feedback provided. Feedback components of task, performance gap and action plan provided by tutors were often identified in the low achieving-students compared to the higher achieving-students in both second and third-years. The decreased identification of these deliberate practice elements in the feedback with increasing level of achievement is attributed to students having no or fewer gaps and hence a decreased need for action plans. Tutor’s cognitive resources and energy was hence directed to the lower-achieving students who needed more of his/her attention. This is similar to other studies in clinical practice where increasing student achievement better directs supervisor’s cognitive resources to patient care instead of educational assessment on a single skill [5]. Advanced learners require feedback focusing more on higher-order integrated learning tasks such as problem solving and clinical reasoning [1].

Specific task was the most frequent component provided to our second-year higher-achieving students as compared to the gap and action feedback component. A reason that may explain this is that the task is the easiest to describe by simply recording a detailed account of the task performed while feedback on the gap and action may be low because the students are performing at a competent level to which they are being evaluated and the feedback instrument may be used primarily to identify competency gaps rather than promoting expertise development. In contrast, tutors focus on the knowledge gap and action plan of students who perform poorly, instead of spending time describing the event.

An overall trend is apparent when comparing student achievement category with the average task gap and action scores in peer feedback. With increasing student achievement, the task, gap and action scores increase, opposite to what we found with the tutor feedback. There is the possibility of peers tending to over-rate the work of their peers so as not to appear too critical and may explain why sometimes students’ lack confidence in their peer’s feedback. Though studies confirm tutor-student feedback dialogue as essential for learning enhancement with tutors perceived as authoritative feedback source and the best person to scaffold student learning [33, 39], Orsmond and Merry in their investigation of high- and low- achieving third-year biology students’ perceptions of teacher feedback, indicated potential disadvantages when teachers are the sole source of feedback input [40]. The low-achieving students depended highly on the teacher to make improvements in their work by consistently focusing on the surface features of feedback messages compared to the high-achieving students who try to seek the meaning behind the feedback message [41]. Nicol suggested peer feedback processes be strengthened for weaker students as peers generating and receiving feedback in relation to the same assessment task learn not only about their own work but also about how it compares with the work of other students [34].

The study has demonstrated an improvement in the written feedback provided to students in clinical skills. Tutors previously provided general comments which were vague and inconsistent [22]. The implementation of a structured feedback improvement strategy encouraged tutors to provide timely and balanced feedback. However despite this intervention there was high variability with regards to specific description of each component as indicated by the low component average scores (2 or < 2). Using the feedback scoring system has also allowed us to identify tutors providing particularly low quality written feedback and hence the need for individualised faculty feedback and development.

An interesting finding in our study was tutor’s provision of global rating on student’s performance of ‘competent’ or ‘superior performance’ with no ‘failure’ suggesting difficulty giving negative feedback. Possible reasons are either tutors don’t want to hurt student’s feelings as this can damage their relationship or the fact that remediation may not be available [42]. Previous studies have reported feedback comments failing to distinguish the competence level of learners [43]. However in this study we found an association between the global rating and quality of feedback. Therefore tutors who tend to put time and thought into providing meaningful comments may also be accurately assessing the performance level of the learner.

Clinical tutors may not be hostile to providing useful feedback but working in an environment that limits their opportunity to do so may explain the low quality of feedback especially in heterogeneous diverse settings. The increasing class population and shortage of tutors necessitated the need to capitalise on peer feedback which has had significant benefits by having different feedback providers commenting on different clinical skills providing students with multiple perspectives as well as multiple opportunities for scaffolding their learning [33].

## Limitations

The study measured the elements of deliberate practice in written feedback, it is however possible that tutors provided more feedback orally to students and this could underestimate the extent of deliberate practice components reported.

Though most of the feedback comments were obvious to score, a distinction between certain components was not always clear such as the gap and action components of deliberate practice. It was sometimes difficult to separate the components from a single comment field. For example a student received the comment “remember: auscultation of the precordium for heart sounds after palpating the position of the apex beat”. This could confirm a gap in the student’s knowledge but also using the term “remember” may imply an instruction for changing future behavior. Both raters scored this as a gap of 1 (alluded to the gap) and an action of 3 (specific plan described) though it may not be necessary to separate these two components.

The feedback process depends on various other external factors such as self-assessment, relationship factors, feedback-seeking behavior, self-reflection, feedback source credibility [11, 20] which were not measured as in this study we only focused on the components of deliberate practice described by Ericsson [13].

## Conclusion and recommendations

The introduction of a feedback improvement strategy to the logbooks increased the quality of the feedback provided as the task, gap and action plans were all included. Formal feedback quality assessment using the deliberate practice framework fosters reflections about the quality of feedback provided and hence its usefulness. Based on the findings of this study we suggest that providing clinical tutors and peers with a feedback-scoring tool to review and score their own feedback for the presence of features of high-quality feedback is likely to guide them to give good quality feedback enhancing their feedback skills [1, 44]. Faculty development to improve delivery of quality feedback is important but not sufficient. Possible reasons as to why quality of feedback remains a challenge might be because focus continues to be on how clinical tutors should construct and deliver feedback, rather than how students receive, respond and use feedback along with creating learning environments with individual follow-up feed-forward improvement plans. Investing in the development of peer assessment and feedback skills is of valuable resource in resource constrained and diverse educational settings enhancing student’s engagement with feedback, self-reflection, self-assessment, development of assessment literacy and self-regulated learning skills that are necessary throughout their clinical career [33]. Hence to overcome barriers to meaningful feedback both institutional and individual efforts are required.

While poor quality feedback is a common problem, this study was conducted in a simulated clinical environment hence caution needs to be taken while generalizing our results to other specialties. This study will however serve as a useful theoretical guide to the planning and evaluation of feedback interventions that would be useful for educational purposes.

## Additional files

Additional file 1: 2nd year clinical skills logbook. (DOC 109 kb)

Additional file 2: 3rd year clinical skills logbook. (DOC 144 kb)

## Abbreviations

AA: Average Achievers
HA: High Achievers
LA: Lower Achievers
NRMSM: Nelson R Mandela School of Medicine
OSCE: Objective Structured Clinical Examination
TGA: Task, Gap and Action
UKZN: University of KwaZulu-Natal

## References

[1] Griffiths JM, Luhanga U, McEwen LA, Schultz K, Dalgarno N (2016). Promoting high-quality feedback. Tool for reviewing feedback given to learners by teachers. Can Fam Physician.

[2] Wright S, Jenkins-Guarnieri M (2012). Student evaluations of teaching: combining the meta-analyses and demonstrating further evidence for effective use. Asses Eval Higher Educ.

[3] van de Ridder JM, Stokking KM, McGaghie WC, Ten Cate OT (2008). What is feedback in clinical education?. Med Educ.

[4] Ende J (1983). Feedback in clinical medical education. JAMA..

[5] Gauthier S, Cavalcanti R, Goguen J, Sibbald M (2015). Deliberate practice as a framework for evaluating feedback in residency training. Med Teach..

[6] García-Sanpedro MJ (2012). Feedback and feedforward: focal points for improving academic performance. Jot se.

[7] Bing-You RG, Trowbridge RL (2009). Why medical educators may be failing at feedback. JAMA..

[8] Kluger A, Van Dijk D (2010). Feedback, the various tasks of the doctor, and the feedforward alternative. Med Educ.

[9] Milan FB, Dyche L, Fletcher J (2011). “How am I doing?” teaching medical students to elicit feedback during their clerkships. Med Teach..

[10] Anderson PA (2012). Giving feedback on clinical skills: are we starving our young?. J Grad Med Educ.

[11] Archer JC (2010). State of the science in health professional education: effective feedback. Med Educ.

[12] Ericsson KA (2004). Deliberate practice and the acquisition and maintenance of expert performance in medicine and related domains. Acad Med.

[13] Ericsson KA (2008). Deliberate practice and acquisition of expert performance: a general overview. AEM..

[14] Krackov SK, Pohl H (2011). Building expertise using the deliberate practice curriculum- planning model. Med Teach..

[15] Veloski JJR, Boex MJ, Grasberger A, Evans DB, Wolfson DB (2006). Systematic review of the literature on assessment, feedback and Physician’s clinical performance: BEME guide no 7. Med Teach..

[16] Heiman HL, Uchida T, Adams C (2012). E-learning and deliberate practice for oral case presentation skills: a randomized trial. Med Teach..

[17] Duvivier R, van Dalen J, Muijtjens A, Moulaert V, van der Vleuten C, Scherpbier A (2011). The role of deliberate practice in the acquisition of clinical skills. BMC Med Educ..

[18] De Beer M, Martensson L (2015). Feedback on students’ clinical reasoning skills during fieldwork education. Aust Occup Ther J.

[19] Jackson JL, Kay C, Jackson WC, Frank M (2015). The quality of written feedback by attendings of internal medicine residents. J Gen Intern Med.

[20] Schartel SA (2012). Giving feedback – an integral part of education. Best Pract Res Clin Anaesthesiol.

[21] Al-Mously N, Nabil NM, Al-Babtain SA, Fouad Abbas MA (2014). Undergraduate medical students’ perceptions on the quality of feedback received during clinical rotations. Med Teach..

[22] Abraham RM, Singaram VS (2016). Third-year medical students’ and clinical teachers’ perceptions of formative assessment feedback in the simulated clinical setting. Afr J Health Professions Educ.

[23] Pronovost PJ, Hudson DW (2012). Improving healthcare quality through organizational peer-to-peer assessment: lessons from the nuclear power industry. BMJ Qual Saf.

[24] Holmboe ES, Hawkins RE, Holmboe ES, Hawkins RE (2008). Constructing an evaluation system for an educational program. Practical guide to the evaluation of clinical competence.

[25] Pelgrim EAM, Kramer AWM, Mokkink HGA, Van der Vleuten CPM (2013). Reflection as a component of formative assessment appears to be instrumental in promoting the use of feedback: an observational study. Med Teach..

[26] Frank JR, Snell L, Sherbino J (2015). CanMEDS 2015 Physician Competency Framework.

[27] Schüttpelz-Brauns K, Narciss E, Schneyinck C (2016). Twelve tips for successfully implementing logbooks in clinical training. Med Teach.

[28] Barr EM (1987). The relationship between student and clinical supervisor. BJ Occup Ther.

[29] Krosnick JA, Pressure S, Marsden PV, Wright JD (2010). Question and questionnaire design. Handbook of survey research (2nd ed., pp. 263–313).

[30] Bernardin HJ, Smith PC (1981). A clarification of some issues regarding the development and use of behaviorally anchored rating scales (BARS). J Appl Psych.

[31] Landis JR, Koch GG (1977). The measurement of observer agreement for categorical data. Biometrics.

[32] Halman S, Dudek N, Wood T (2016). direct observation of clinical skills feedback scale: development and validity evidence. Teach Learn Med.

[33] Falchikov N (2005). Improving assessment through student involvement.

[34] Nicol DJ (2010). From monologue to dialogue: improving written feedback processes in mass higher education. Assess Eval Higher Educ.

[35] Cantillon P, Sargeant J (2008). Giving feedback in clinical settings. BMJ..

[36] Bok HG, Teunissen PW, Favier RP (2013). Programmatic assessment of competency- based workplace learning: when theory meets practice. BMC Med Educ.

[37] Bates J, Konkin J, Suddards C, Dobson S, Pratt D (2013). Student perceptions of assessment and feedback in longitudinal integrated clerkships. Med Educ.

[38] Telio S, Ajjawi R, Regehr G (2015). The “educational Alliance” as a framework for reconceptualizing feedback in medical education. Acad Med.

[39] Chi MT, Roy HM, Hausmann RGM (2008). Observing dialogues collaboratively: insights about human tutoring effectiveness from vicarious learning. Cogn Sci.

[40] Merry Stephen, Orsmond Paul (2008). Students’ Attitudes to and Usage of Academic Feedback Provided Via Audio Files. Bioscience Education.

[41] Dunworth K, Sanchez HS (2016). Perceptions of quality in staff-student written feedback in higher education: a case study. Teach Higher Educ.

[42] Daelmans HEM, Overmeer RM (2006). Van der hem-Stokroos HH, Scherpbier AJJA, Stehouwer CDA, van der Vleuten CPM. In-training assessment: qualitative study of effects on supervision and feedback in an undergraduate clinical rotation. Med Educ.

[43] Hawkins RE, Sumption KF, Gaglione MM, Holmboe ES (1999). The in-training examination in internal medicine: resident perceptions and lack of correlation between resident scores and faculty predictions of resident performance. Am J Med.

[44] Iobst WF, Sherbino J, ten Cate O, Richardson DL, Dath D, Swing SR (2010). Competency based medical education in postgraduate medical education. Med Teach..

