# Supplementary material for: Using deliberate practice framework to assess the quality of feedback in undergraduate clinical skills training
Source: BMC Med Educ. 2019 Apr 11;19:105. doi: 10.1186/s12909-019-1547-5 (PMC6460682; doi:10.1186/s12909-019-1547-5)
Supplement: Supplementary file 1 — 2nd year clinical skills logbook. (DOC 109 kb) [file 12909_2019_1547_MOESM1_ESM.doc]

**CLINICAL SKILLS**

**MBChB 2**

**2018**

**Clinical Skills**

**Logbook**

**Students’ Copy**

**Updated December 2017**

**Student Name**: ……………………………………………………………….

**Student Number**: …………………………………………………………….

**Contents**

1. Instructions and Notes to Students
2. Summary Page
3. Forms for completion

**Instructions and Notes to Students**

This clinical skills logbook has been designed to reinforce your knowledge of and ability in certain examination and procedural skills, and to improve your confidence in examining patients as you approach your clinical years. Only a few key skills have been identified, which are particularly important for you to master as soon as possible.

There are several skills in the logbook. During the course of the year, each of you will be required to perform certain skills satisfactorily in the presence of one of the clinicians or Skills Lab staff in order for these to be signed off.

**The listed skills are:**

1) General examination

2) Taking a pulse and measuring blood pressure

3) Examination of the JVP and praecordium

4) Examination of the chest

5) Examination of the abdomen

6) All procedural skills taught during each theme

Times will be made available in some themes, and you will need to be present at these sessions for assessment. You will be given 8 minutes to carry out the skill, demonstrating it once sequentially in this time. A student who fails to perform the examination successfully in the session will be asked to repeat the session, at least a week later, to ensure that s/he revises and practises adequately in preparation. In this case, you will need to make a special arrangement with one of the clinicians to assess you in a lunch hour or on a Saturday, subject to availability, and provide a patient for the session. Each skill may only be examined twice. Students who do not attend in a booked repeat slot (which is not cancelled at least the day before) will be marked as unsuccessful for that skill.

Completion of the logbooks is a DP requirement, and they must be handed in by a date to be announced. For this reason, please make sure to practise and book your slots timeously. In terms of performance, there are 4 zones: zone of failure, weak pass, competence or superior performance. You will need to be marked as (at least) *Competent* in all scheduled logbook sessions during the course of the year. Please do not lose your logbooks, as these are your proof of satisfactory completion. Note also that no pages may be removed from the logbook under any circumstances.

These assessments are intended to be formative, but are not teaching sessions. Each examiner will have available a list of minimum requirements for the skill to be deemed to have been performed satisfactorily. These are **not** OSCE checklists, but are considered to be the **minimum** requirements for a student to demonstrate competence in continuous assessment at MBChB 2 level. If core competencies are missing or unreliable, performance is rated as *Failure***,** and the student should re-book an assessment as described above. (Written feedback will be provided to guide you in your learning). If the student’s performance within the allocated time demonstrates a confident technique, with good knowledge and understanding of the clinical skill, s/he will be rated as Superior performance.

Though you would not be given a grade, in summary the zones are weighted accordingly for your learning experience:

Zone of failure relates to <48%

Core competencies are missing or unreliable

Zone of competence relates to 60%

Competent pass

Zone of superior performance relates to 80%

Confident technique

Good knowledge and understanding

Note that we will be assessing you as an MBChB 2 student. Please remember that examination and procedural skills require ongoing repetition in order to master techniques, to continue to improve and to reach the level of competence expected of you in the clinical years and in practice. Thus, even if your skill is marked as competent or superior performance for 2^nd^ Year, there is much further improvement expected.

**Please note:**

**General requirements of students in the logbook sessions include the following, but you will be guided by your examiner:**

1. Attends well presented, appropriately dressed in a **clean and ironed** white coat with gloves and stethoscope
2. Greets patient professionally (introduces him- or herself and obtains patient’s name), explains nature of examination/ procedure and obtains consent
3. Mentions privacy, positions patient correctly and comfortably, and exposes him/her correctly (according to nature of examination/ procedure)
4. Mentions focused general examination (where relevant)
5. Performs the examination/ procedure in an appropriate and logical sequence
6. Completes all important parts of the relevant examination/ procedure and demonstrates correct technique.
7. Explains correctly to and shows the patient what is required of him/ her during the examination/ procedure
8. Treats patient courteously and gently throughout the examination/ procedure, informs him/her of the findings, and thanks and makes patient comfortable on completion e.g “Thank you, Mrs Singh – your pulse and blood pressure are normal.”
9. Uses correct terminology when explaining his/ her actions and findings to the examiner.

**10) Briefly** summarises findings related to the examination to the examiner e.g “The JVP was normal. Examination of the praecordium was normal, with a heart rate of 72, normal S1 and S2 and no murmurs noted.”

I hope that this will be a useful exercise and look forward to assisting you improve your clinical skills. Please remember to refer to your Clinical Skills resource material, including that available on the LAN. Good luck (and much practice!) to you all!

**December 2017**

**Summary Page**

**Student Name**: ……………………………………………………………….

**Student Number**: …………………………………………………………….

**List of Examination Skills:**

**DATE COMPLETED SATISFACTORILY**

1. **General examination……………………………………………………………………..**
2. **Taking a pulse and measuring blood pressure……………………………………….......................................................**
3. **Examination of the JVP and praecordium………………………………………………………………………………..**
4. **Examination of the chest………………………………………………………………….**
5. **Examination of the abdomen……………………………………………………………..**

**List of Procedural Skills:**

**1) Asthma adjuncts………………………………………………………………………….**

**2) Chest X-ray………………………………………………………………………………...**

**3) ECG Strip interpretation………………………………………………………………...**

**4) Urinalysis…………………………………………………………………………………..**

**5) Urethral Catheterisation…………………………………………………………………**

**6) Body mass index and Hip to waist circumference…...........................................**

**7) Biceps and Triceps skin fold thickness measurement……………………………**

**8) Examination of an Abdominal mass………………………………………………….**

**9) Abdominal X-ray…………………………………………………………………………**

**10) Examination of the Newborn………………………………………………………….**

**11) Examination of the Thyroid gland…………………………………………………..**

**12) Intravenous cannulation………………………………………………………………**

**13) Rapid glucose and cholesterol measurement…………………………………….**

**THEME 2.1**

**ASSESSMENT OF CLINICAL EXAMINATION SKILLS**

**SKILL ASSESSED:_________________________________________________________**

**ASSESSED BY:____________________________________________________________**

**DATE:____________________________________________________________________**

**ZONE OF PERFORMANCE (MBChB 2 level):**

| **FAILURE** | **WEAK PASS** | **COMPETENT** | **SUPERIOR**  **PERFORMANCE** |
| --- | --- | --- | --- |

**COMMENTS:**

**1) WHAT WAS DONE WELL?**

**_________________________________________________________________________**

**_________________________________________________________________________**

**2) WHAT WAS NOT DONE WELL?**

**___________________________________________________________________________________________________________________________________________________________________________________________________________________________**

**3) WHAT CAN BE IMPROVED?**

**____________________________________________________________________________________________________________________________________________________________________________________________________________________________________________________________________________________________________**

**SIGNED:___________________**

**REPEAT ASSESSMENT:**

**ASSESSED BY:____________________________________________________________**

**DATE:____________________________________________________________________**

**ZONE OF PERFORMANCE (MBChB 2 level):**

| **FAILURE** | **WEAK PASS** | **COMPETENT** | **SUPERIOR**  **PERFORMANCE** |
| --- | --- | --- | --- |

**COMMENTS:**

**1) WHAT WAS DONE WELL?**

**______________________________________________________________**

**_________________________________________________________________________**

**2) WHAT WAS NOT DONE WELL?**

**___________________________________________________________________________________________________________________________________________________________________________________________________________________________**

**3) WHAT CAN BE IMPROVED?**

**____________________________________________________________________________________________________________________________________________________________________________________________________________________________________________________________________________________________________**

**SIGNED:___________________**

**PROCEDURAL SKILLS**

**ASTHMA ADJUNCTS:**

**ASSESSED BY:____________________________________________________________**

**DATE:____________________________________________________________________**

**ZONE OF PERFORMANCE (MBChB 2 level):**

| **FAILURE** | **WEAK PASS** | **COMPETENT** | **SUPERIOR**  **PERFORMANCE** |
| --- | --- | --- | --- |

**COMMENTS:**

**1) WHAT WAS DONE WELL?**

**______________________________________________________________**

**_________________________________________________________________________**

**2) WHAT WAS NOT DONE WELL?**

**___________________________________________________________________________________________________________________________________________________________________________________________________________________________**

**3) WHAT CAN BE IMPROVED?**

**____________________________________________________________________________________________________________________________________________________________________________________________________________________________________________________________________________________________________**

**SIGNED:___________________**

**SYSTEMATIC APPROACH TO INTERPRETATION OF A NORMAL CHEST X-RAY:**

**ASSESSED BY:____________________________________________________________**

**DATE:____________________________________________________________________**

**ZONE OF PERFORMANCE (MBChB 2 level):**

| **FAILURE** | **WEAK PASS** | **COMPETENT** | **SUPERIOR**  **PERFORMANCE** |
| --- | --- | --- | --- |

**COMMENTS:**

**1) WHAT WAS DONE WELL?**

**______________________________________________________________**

**_________________________________________________________________________**

**2) WHAT WAS NOT DONE WELL?**

**___________________________________________________________________________________________________________________________________________________________________________________________________________________________**

**3) WHAT CAN BE IMPROVED?**

**____________________________________________________________________________________________________________________________________________________________________________________________________________________________________________________________________________________________________**

**SIGNED:___________________**

**THEME 2.2**

**ASSESSMENT OF CLINICAL EXAMINATION SKILLS**

**SKILL ASSESSED:_________________________________________________________**

**ASSESSED BY:____________________________________________________________**

**DATE:____________________________________________________________________**

**ZONE OF PERFORMANCE (MBChB 2 level):**

| **FAILURE** | **WEAK PASS** | **COMPETENT** | **SUPERIOR**  **PERFORMANCE** |
| --- | --- | --- | --- |

**COMMENTS:**

**1) WHAT WAS DONE WELL?**

**_________________________________________________________________________**

**_________________________________________________________________________**

**2) WHAT WAS NOT DONE WELL?**

**___________________________________________________________________________________________________________________________________________________________________________________________________________________________**

**3) WHAT CAN BE IMPROVED?**

**____________________________________________________________________________________________________________________________________________________________________________________________________________________________________________________________________________________________________**

**SIGNED:___________________**

**REPEAT ASSESSMENT:**

**ASSESSED BY:____________________________________________________________**

**DATE:____________________________________________________________________**

**ZONE OF PERFORMANCE (MBChB 2 level):**

| **FAILURE** | **WEAK PASS** | **COMPETENT** | **SUPERIOR**  **PERFORMANCE** |
| --- | --- | --- | --- |

**COMMENTS:**

**1) WHAT WAS DONE WELL?**

**______________________________________________________________**

**_________________________________________________________________________**

**2) WHAT WAS NOT DONE WELL?**

**___________________________________________________________________________________________________________________________________________________________________________________________________________________________**

**3) WHAT CAN BE IMPROVED?**

**____________________________________________________________________________________________________________________________________________________________________________________________________________________________________________________________________________________________________**

**SIGNED:___________________**

**PROCEDURAL SKILLS**

**SYSTEMATIC APPROACH TO INTERPRETATION OF A NORMAL ECG STRIP:**

**ASSESSED BY:____________________________________________________________**

**DATE:____________________________________________________________________**

**ZONE OF PERFORMANCE (MBChB 2 level):**

| **FAILURE** | **WEAK PASS** | **COMPETENT** | **SUPERIOR**  **PERFORMANCE** |
| --- | --- | --- | --- |

**COMMENTS:**

**1) WHAT WAS DONE WELL?**

**______________________________________________________________**

**_________________________________________________________________________**

**2) WHAT WAS NOT DONE WELL?**

**___________________________________________________________________________________________________________________________________________________________________________________________________________________________**

**3) WHAT CAN BE IMPROVED?**

**____________________________________________________________________________________________________________________________________________________________________________________________________________________________________________________________________________________________________**

**SIGNED:___________________**

**THEME 2.3**

**ASSESSMENT OF CLINICAL EXAMINATION SKILLS**

**SKILL ASSESSED:_________________________________________________________**

**ASSESSED BY:____________________________________________________________**

**DATE:____________________________________________________________________**

**ZONE OF PERFORMANCE (MBChB 2 level):**

| **FAILURE** | **WEAK PASS** | **COMPETENT** | **SUPERIOR**  **PERFORMANCE** |
| --- | --- | --- | --- |

**COMMENTS:**

**1) WHAT WAS DONE WELL?**

**_________________________________________________________________________**

**_________________________________________________________________________**

**2) WHAT WAS NOT DONE WELL?**

**___________________________________________________________________________________________________________________________________________________________________________________________________________________________**

**3) WHAT CAN BE IMPROVED?**

**____________________________________________________________________________________________________________________________________________________________________________________________________________________________________________________________________________________________________**

**SIGNED:___________________**

**REPEAT ASSESSMENT:**

**ASSESSED BY:____________________________________________________________**

**DATE:____________________________________________________________________**

**ZONE OF PERFORMANCE (MBChB 2 level):**

| **FAILURE** | **WEAK PASS** | **COMPETENT** | **SUPERIOR**  **PERFORMANCE** |
| --- | --- | --- | --- |

**COMMENTS:**

**1) WHAT WAS DONE WELL?**

**______________________________________________________________**

**_________________________________________________________________________**

**2) WHAT WAS NOT DONE WELL?**

**___________________________________________________________________________________________________________________________________________________________________________________________________________________________**

**3) WHAT CAN BE IMPROVED?**

**____________________________________________________________________________________________________________________________________________________________________________________________________________________________________________________________________________________________________**

**SIGNED:___________________**

**PROCEDURAL SKILLS**

**URINALYSIS:**

**SKILL ASSESSED:_________________________________________________________**

**ASSESSED BY:____________________________________________________________**

**DATE:____________________________________________________________________**

**ZONE OF PERFORMANCE (MBChB 2 level):**

| **FAILURE** | **WEAK PASS** | **COMPETENT** | **SUPERIOR**  **PERFORMANCE** |
| --- | --- | --- | --- |

**COMMENTS:**

**1) WHAT WAS DONE WELL?**

**_________________________________________________________________________**

**_________________________________________________________________________**

**2) WHAT WAS NOT DONE WELL?**

**___________________________________________________________________________________________________________________________________________________________________________________________________________________________**

**3) WHAT CAN BE IMPROVED?**

**____________________________________________________________________________________________________________________________________________________________________________________________________________________________________________________________________________________________________**

**SIGNED:___________________**

**MALE AND FEMALE URETHRAL CATHETERISATION:**

**ASSESSED BY:____________________________________________________________**

**DATE:____________________________________________________________________**

**ZONE OF PERFORMANCE (MBChB 2 level):**

| **FAILURE** | **WEAK PASS** | **COMPETENT** | **SUPERIOR**  **PERFORMANCE** |
| --- | --- | --- | --- |

**COMMENTS:**

**1) WHAT WAS DONE WELL?**

**______________________________________________________________**

**_________________________________________________________________________**

**2) WHAT WAS NOT DONE WELL?**

**___________________________________________________________________________________________________________________________________________________________________________________________________________________________**

**3) WHAT CAN BE IMPROVED?**

**____________________________________________________________________________________________________________________________________________________________________________________________________________________________________________________________________________________________________**

**SIGNED:___________________**

**THEME 2.4**

**ASSESSMENT OF CLINICAL EXAMINATION SKILLS**

**SKILL ASSESSED:_________________________________________________________**

**ASSESSED BY:____________________________________________________________**

**DATE:____________________________________________________________________**

**ZONE OF PERFORMANCE (MBChB 2 level):**

| **FAILURE** | **WEAK PASS** | **COMPETENT** | **SUPERIOR**  **PERFORMANCE** |
| --- | --- | --- | --- |

**COMMENTS:**

**1) WHAT WAS DONE WELL?**

**_________________________________________________________________________**

**_________________________________________________________________________**

**2) WHAT WAS NOT DONE WELL?**

**___________________________________________________________________________________________________________________________________________________________________________________________________________________________**

**3) WHAT CAN BE IMPROVED?**

**____________________________________________________________________________________________________________________________________________________________________________________________________________________________________________________________________________________________________**

**SIGNED:___________________**

**REPEAT ASSESSMENT:**

**ASSESSED BY:____________________________________________________________**

**DATE:____________________________________________________________________**

**ZONE OF PERFORMANCE (MBChB 2 level):**

| **FAILURE** | **WEAK PASS** | **COMPETENT** | **SUPERIOR**  **PERFORMANCE** |
| --- | --- | --- | --- |

**COMMENTS:**

**1) WHAT WAS DONE WELL?**

**______________________________________________________________**

**_________________________________________________________________________**

**2) WHAT WAS NOT DONE WELL?**

**___________________________________________________________________________________________________________________________________________________________________________________________________________________________**

**3) WHAT CAN BE IMPROVED?**

**____________________________________________________________________________________________________________________________________________________________________________________________________________________________________________________________________________________________________**

**SIGNED:___________________**

**PROCEDURAL SKILLS**

**BODY MASS INDEX AND HIP TO WAIST CIRCUMFERENCE MEASUREMENTS:**

**ASSESSED BY:____________________________________________________________**

**DATE:____________________________________________________________________**

**ZONE OF PERFORMANCE (MBChB 2 level):**

| **FAILURE** | **WEAK PASS** | **COMPETENT** | **SUPERIOR**  **PERFORMANCE** |
| --- | --- | --- | --- |

**COMMENTS:**

**1) WHAT WAS DONE WELL?**

**______________________________________________________________**

**_________________________________________________________________________**

**2) WHAT WAS NOT DONE WELL?**

**___________________________________________________________________________________________________________________________________________________________________________________________________________________________**

**3) WHAT CAN BE IMPROVED?**

**____________________________________________________________________________________________________________________________________________________________________________________________________________________________________________________________________________________________________**

**SIGNED:___________________**

**BICEPS AND TRICEPS SKIN FOLD THICKNESS MEASUREMENTS:**

**ASSESSED BY:____________________________________________________________**

**DATE:____________________________________________________________________**

**ZONE OF PERFORMANCE (MBChB 2 level):**

| **FAILURE** | **WEAK PASS** | **COMPETENT** | **SUPERIOR**  **PERFORMANCE** |
| --- | --- | --- | --- |

**COMMENTS:**

**1) WHAT WAS DONE WELL?**

**______________________________________________________________**

**_________________________________________________________________________**

**2) WHAT WAS NOT DONE WELL?**

**___________________________________________________________________________________________________________________________________________________________________________________________________________________________**

**3) WHAT CAN BE IMPROVED?**

**____________________________________________________________________________________________________________________________________________________________________________________________________________________________________________________________________________________________________**

**SIGNED:___________________**

**ASSESSMENT OF EXAMINATION OF AN ABDOMINAL MASS**

**SKILL ASSESSED:_________________________________________________________**

**ASSESSED BY:____________________________________________________________**

**DATE:____________________________________________________________________**

**ZONE OF PERFORMANCE (MBChB 2 level):**

| **FAILURE** | **WEAK PASS** | **COMPETENT** | **SUPERIOR**  **PERFORMANCE** |
| --- | --- | --- | --- |

**COMMENTS:**

**1) WHAT WAS DONE WELL?**

**_________________________________________________________________________**

**_________________________________________________________________________**

**2) WHAT WAS NOT DONE WELL?**

**___________________________________________________________________________________________________________________________________________________________________________________________________________________________**

**3) WHAT CAN BE IMPROVED?**

**____________________________________________________________________________________________________________________________________________________________________________________________________________________________________________________________________________________________________**

**SIGNED:___________________**

**SYSTEMATIC APPROACH TO INTERPRETATION OF A NORMAL ABDOMINAL X-RAY:**

**ASSESSED BY:____________________________________________________________**

**DATE:____________________________________________________________________**

**ZONE OF PERFORMANCE (MBChB 2 level):**

| **FAILURE** | **WEAK PASS** | **COMPETENT** | **SUPERIOR**  **PERFORMANCE** |
| --- | --- | --- | --- |

**COMMENTS:**

**1) WHAT WAS DONE WELL?**

**______________________________________________________________**

**_________________________________________________________________________**

**2) WHAT WAS NOT DONE WELL?**

**___________________________________________________________________________________________________________________________________________________________________________________________________________________________**

**3) WHAT CAN BE IMPROVED?**

**____________________________________________________________________________________________________________________________________________________________________________________________________________________________________________________________________________________________________**

**SIGNED:___________________**

**THEME 2.5**

**ASSESSMENT OF CLINICAL EXAMINATION SKILLS**

**SKILL ASSESSED:_________________________________________________________**

**ASSESSED BY:____________________________________________________________**

**DATE:____________________________________________________________________**

**ZONE OF PERFORMANCE (MBChB 2 level):**

| **FAILURE** | **WEAK PASS** | **COMPETENT** | **SUPERIOR**  **PERFORMANCE** |
| --- | --- | --- | --- |

**COMMENTS:**

**1) WHAT WAS DONE WELL?**

**_________________________________________________________________________**

**_________________________________________________________________________**

**2) WHAT WAS NOT DONE WELL?**

**___________________________________________________________________________________________________________________________________________________________________________________________________________________________**

**3) WHAT CAN BE IMPROVED?**

**____________________________________________________________________________________________________________________________________________________________________________________________________________________________________________________________________________________________________**

**SIGNED:___________________**

**REPEAT ASSESSMENT:**

**ASSESSED BY:____________________________________________________________**

**DATE:____________________________________________________________________**

**ZONE OF PERFORMANCE (MBChB 2 level):**

| **FAILURE** | **WEAK PASS** | **COMPETENT** | **SUPERIOR**  **PERFORMANCE** |
| --- | --- | --- | --- |

**COMMENTS:**

**1) WHAT WAS DONE WELL?**

**______________________________________________________________**

**_________________________________________________________________________**

**2) WHAT WAS NOT DONE WELL?**

**___________________________________________________________________________________________________________________________________________________________________________________________________________________________**

**3) WHAT CAN BE IMPROVED?**

**____________________________________________________________________________________________________________________________________________________________________________________________________________________________________________________________________________________________________**

**SIGNED:___________________**

**ASSESSMENT OF EXAMINATION OF THE NEWBORN**

**SKILL ASSESSED:_________________________________________________________**

**ASSESSED BY:____________________________________________________________**

**DATE:____________________________________________________________________**

**ZONE OF PERFORMANCE (MBChB 2 level):**

| **FAILURE** | **WEAK PASS** | **COMPETENT** | **SUPERIOR**  **PERFORMANCE** |
| --- | --- | --- | --- |

**COMMENTS:**

**1) WHAT WAS DONE WELL?**

**_________________________________________________________________________**

**_________________________________________________________________________**

**2) WHAT WAS NOT DONE WELL?**

**___________________________________________________________________________________________________________________________________________________________________________________________________________________________**

**3) WHAT CAN BE IMPROVED?**

**____________________________________________________________________________________________________________________________________________________________________________________________________________________________________________________________________________________________________**

**SIGNED:___________________**

**ASSESSMENT OF THYROID EXAMINATION SKILLS**

**SKILL ASSESSED:_________________________________________________________**

**ASSESSED BY:____________________________________________________________**

**DATE:____________________________________________________________________**

**ZONE OF PERFORMANCE (MBChB 2 level):**

| **FAILURE** | **WEAK PASS** | **COMPETENT** | **SUPERIOR**  **PERFORMANCE** |
| --- | --- | --- | --- |

**COMMENTS:**

**1) WHAT WAS DONE WELL?**

**_________________________________________________________________________**

**_________________________________________________________________________**

**2) WHAT WAS NOT DONE WELL?**

**___________________________________________________________________________________________________________________________________________________________________________________________________________________________**

**3) WHAT CAN BE IMPROVED?**

**____________________________________________________________________________________________________________________________________________________________________________________________________________________________________________________________________________________________________**

**SIGNED:___________________**

**PROCEDURAL SKILLS**

**INTRAVENOUS CANNULATION:**

**ASSESSED BY:____________________________________________________________**

**DATE:____________________________________________________________________**

**ZONE OF PERFORMANCE (MBChB 2 level):**

| **FAILURE** | **WEAK PASS** | **COMPETENT** | **SUPERIOR**  **PERFORMANCE** |
| --- | --- | --- | --- |

**COMMENTS:**

**1) WHAT WAS DONE WELL?**

**______________________________________________________________**

**_________________________________________________________________________**

**2) WHAT WAS NOT DONE WELL?**

**___________________________________________________________________________________________________________________________________________________________________________________________________________________________**

**3) WHAT CAN BE IMPROVED?**

**____________________________________________________________________________________________________________________________________________________________________________________________________________________________________________________________________________________________________**

**SIGNED:___________________**

**RAPID GLUCOSE AND CHOLESTEROL MEASUREMENT:**

**ASSESSED BY:____________________________________________________________**

**DATE:____________________________________________________________________**

**ZONE OF PERFORMANCE (MBChB 2 level):**

| **FAILURE** | **WEAK PASS** | **COMPETENT** | **SUPERIOR**  **PERFORMANCE** |
| --- | --- | --- | --- |

**COMMENTS:**

**1) WHAT WAS DONE WELL?**

**_________________________________________________________________________**

**_________________________________________________________________________**

**2) WHAT WAS NOT DONE WELL?**

**___________________________________________________________________________________________________________________________________________________________________________________________________________________________**

**3) WHAT CAN BE IMPROVED?**

**____________________________________________________________________________________________________________________________________________________________________________________________________________________________________________________________________________________________________**

**SIGNED:___________________**
