# Supplementary material for: Using deliberate practice framework to assess the quality of feedback in undergraduate clinical skills training
Source: BMC Med Educ. 2019 Apr 11;19:105. doi: 10.1186/s12909-019-1547-5 (PMC6460682; doi:10.1186/s12909-019-1547-5)
Supplement: Supplementary file 2 — 3rd year clinical skills logbook. (DOC 144 kb) [file 12909_2019_1547_MOESM2_ESM.doc]

**CLINICAL SKILLS**

**MBChB 3**

**2018**

**Clinical Skills**

**Logbook**

**Students’ Copy**

**Updated December 2017**

**Student Name**:……………………………………………………………….

**Student Number**:…………………………………………………………….

**Contents**

1. **Instructions and notes to students**
2. **Summary page for students**
3. **Forms for completion**

**Instructions and Notes to Students**

This logbook has been designed to reinforce your knowledge of and ability in certain clinical skills, and to improve your confidence in examining patients as you approach your clinical years. Key skills have been identified, which are particularly important for you to master as soon as possible.

There are 4 new examination skills in the logbook. During the course of the year, each of which you will be required to perform certain skills satisfactorily in the presence of one of the clinicians or Skills Lab staff in order for these to be signed off.

**The new 3^rd^ Year examination skills for this semester are:**

1) Neuro 1 - motor examination

2) Neuro 2 - sensory examination

3) Neuro 3 - examination of co-ordination

4) Neuro 4 - examination of the cranial nerves

**In addition, you may be called upon to perform your 2^nd^ Year examination skills, as follows:**

1) Detailed examination of the pulses and measurement of BP

2) Examination of the JVP and praecordium (including general exam)

3) Examination of the chest (including general exam)

4) Examination of the abdomen (including general exam)

Times will be made available in some themes, and you will need to be present at these sessions for assessment. You will be given 8 minutes to carry out the skill, demonstrating it once sequentially in this time. A student who fails to perform the examination successfully in the session will be asked to repeat the session, at least a week later, to ensure that s/he revises and practises adequately in preparation. In this case, you will need to make a special arrangement with one of the clinicians to assess you in a lunch hour or on a Saturday, subject to availability, and provide a patient for the session. Each skill may only be examined twice. Students who do not attend in a booked repeat slot (which is not cancelled at least the day before) will be marked as unsuccessful for that skill.

Completion of the logbooks is a DP requirement, and logbooks must be handed in by a date to be announced. For this reason, please make sure to practise and book your slots timeously. In terms of performance, there are 4 zones: zone of failure, weak pass, competence or superior performance. You will need to be marked as (at least) *Competent* in specified logbook sessions during the course of the year. Please do not lose your logbooks, as these are your proof of satisfactory completion. Note also that no pages may be removed from the logbook under any circumstances.

These assessments are intended to be formative, but are not teaching sessions. Each examiner will have available a list of minimum requirements for the skill to be deemed to have been performed satisfactorily. These are **not** OSCE checklists, but are considered to be the minimum requirements for a student who has passed through the MBChB 3 Skills programme. If core competencies are missing or unreliable, performance is rated as *Failure*, and the student should re-book an assessment as described above. (Written feedback will be provided to guide you in your learning). If the student’s performance within the allocated time demonstrates a confident technique with good knowledge and understanding of the clinical skill, s/he will be rated as *Superior performance*.

Though you will not be given a mark, to assist you in understanding your level of mastery of the skill, in summary the zones relate to the following:

**Zone of failure < 48%:**

**Core competencies are missing or unreliable**

**Zone of competence approximately 60%:**

**Competent pass**

**Zone of superior performance approximately 80%:**

**Confident technique**

**Good knowledge and understanding**

Note that we will be assessing you as an MBChB 3 student. Please remember that clinical skills require ongoing repetition in order to master techniques, to continue to improve and to reach the level of competence expected of you in the clinical years and in practice. Thus, even if your skill is marked as satisfactory or above average for 3rd Year, there is much further improvement expected.

**Please note:**

**General requirements of students in the logbook sessions include the following, but be guided by your examiner:**

1. Attends well presented, appropriately dressed in a **clean and ironed** white coat with gloves and stethoscope
2. Greets patient professionally (introduces him- or herself and obtains patient’s name), explains nature of examination/ procedure and obtains consent
3. Mentions privacy, positions patient correctly and comfortably, and exposes him/her correctly (according to nature of examination/ procedure)
4. Mentions focused general examination (where relevant)
5. Performs the examination/ procedure in an appropriate and logical sequence
6. Completes all important parts of the relevant examination/ procedure and demonstrates correct technique.
7. Explains correctly to and shows the patient what is required of him/ her during the examination/ procedure
8. Treats patient courteously and gently throughout the examination/ procedure, informs him/her of the findings, and thanks and makes patient comfortable on completion eg “Thank you, Mrs Pather – your reflexes are normal.”
9. Uses correct terminology when explaining his/ her actions and findings to the examiner

**10)** Briefly summarises findings to the examiner eg “The motor examination was normal, with no wasting, normal tone and power, and reflexes present and equal.”

I hope that this will be a useful exercise and look forward to assisting you as you continue to improve your clinical skills. Please remember to refer to your Clinical Skills resource material, including that available on the LAN, and to keep practising new techniques and revising skills previously acquired. Best wishes to you all for the 3^rd^ Year.

**December 2017**

**Summary Page**

**Student Name**:……………………………………………………………….

**Student Number**:…………………………………………………………….

**List of New Examination Skills:**

**DATE COMPLETED SATISFACTORILY**

1. **Neuro 1 – motor examination……………………………………………………………**
2. **Neuro 2 – sensory examination…………………………………………………………**

**3) Neuro 3 – examination of co-ordination………………………………………………**

1. **Neuro 4 – examination of the cranial nerves…………………………………………**

**List of Revision Skills:**

**1) Detailed examination of pulses and measurement of BP**

**2) Examination of the JVP and praecordium (including general examination)**

**3) Examination of the chest (including general examination)**

**4) Examination of the abdomen (including general examination)**

**List of Procedural Skills:**

1. **Developmental assessment (infant/child)**
2. **Lumbar puncture**
3. **A practical approach to fundoscopy**
4. **A basic approach to X-Rays of the spine, bones and joints**
5. **Gynaecological Examination**
6. **Pap smear**
7. **Obstetric Abdominal exam**
8. **Partogram & Mechanism of labour and delivery**
9. **Examination of the male genitalia and rectal examination**
10. **Bedside haemoglobin test**
11. **Rapid HIV testing**
12. **TB testing in children: Mantoux, sputum collection, gastric washing**
13. **Specimen collection: urine & stool specimens, nasal and throat swabs, sputum specimens, wound specimens, fungal scrape and pus aspirate, genital specimens - male and female, blood culture + blood collection using the vacutainer**
14. **Neonatal resuscitation**
15. **Hand hygiene, gloving and gowning**

**THEME 3.1**

**ASSESSMENT OF CLINICAL EXAMINATION SKILLS**

**SKILL ASSESSED:_________________________________________________________**

**ASSESSED BY:____________________________________________________________**

**DATE:____________________________________________________________________**

**ZONE OF PERFORMANCE (MBChB 3 level):**

| **FAILURE** | **WEAK PASS** | **COMPETENT** | **SUPERIOR**  **PERFORMANCE** |
| --- | --- | --- | --- |

**COMMENTS:**

**1) WHAT WAS DONE WELL?**

**_________________________________________________________________________**

**_________________________________________________________________________**

**2) WHAT WAS NOT DONE WELL?**

**___________________________________________________________________________________________________________________________________________________________________________________________________________________________**

**3) WHAT CAN BE IMPROVED?**

**___________________________________________________________________________________________________________________________________________________________________________________________________________________________**

**SIGNED:___________________**

**REPEAT ASSESSMENT:**

**ASSESSED BY:____________________________________________________________**

**DATE:____________________________________________________________________**

**ZONE OF PERFORMANCE (MBChB 3 level):**

| **FAILURE** | **WEAK PASS** | **COMPETENT** | **SUPERIOR**  **PERFORMANCE** |
| --- | --- | --- | --- |

**COMMENTS:**

**1) WHAT WAS DONE WELL?**

**______________________________________________________________**

**_________________________________________________________________________**

**2) WHAT WAS NOT DONE WELL?**

**___________________________________________________________________________________________________________________________________________________________________________________________________________________________**

**3) WHAT CAN BE IMPROVED?**

**____________________________________________________________________________________________________________________________________________________________________________________________________________________________________________________________________________________________________**

**SIGNED:___________________**

**PROCEDURAL SKILLS**

**DEVELOPMENTAL ASSESSMENT**

**ASSESSED BY:___________________________________________**

**DATE:______________________________________________________**

ZONE OF PERFORMANCE (MBChB 3 level)

| **FAILURE** | **WEAK PASS** | **COMPETENT** | **SUPERIOR**  **PERFORMANCE** |
| --- | --- | --- | --- |

**COMMENTS:**

1. **WHAT WAS DONE WELL?**

**_________________________________________________________________________________________________________________________________________________________________________________________________________**

1. **WHAT WAS NOT DONE WELL?**

**________________________________________________________________________________________________________________________________________________________________________________________________________**

1. **WHAT CAN BE IMPROVED?**

**________________________________________________________________________________________________________________________________________________________________________________________________________**

**SIGNED;____________________________________**

**LUMBAR PUNCTURE:**

**ASSESSED BY:____________________________________________________________**

**DATE:____________________________________________________________________**

**ZONE OF PERFORMANCE (MBChB 3 level):**

| **FAILURE** | **WEAK PASS** | **COMPETENT** | **SUPERIOR**  **PERFORMANCE** |
| --- | --- | --- | --- |

**COMMENTS:**

**1) WHAT WAS DONE WELL?**

**______________________________________________________________**

**_________________________________________________________________________**

**2) WHAT WAS NOT DONE WELL?**

**___________________________________________________________________________________________________________________________________________________________________________________________________________________________**

**3) WHAT CAN BE IMPROVED?**

**____________________________________________________________________________________________________________________________________________________________________________________________________________________________________________________________________________________________________**

**SIGNED:___________________**

**THEME 3.2**

**ASSESSMENT OF CLINICAL EXAMINATION SKILLS**

**SKILL ASSESSED:___________________________________________**

**ASSESSED BY:______________________________________________**

**DATE: ______________________________________________________**

**ZONE OF PERFORMANCE** (MBChB 3 level)

| **FAILURE** | **WEAK PASS** | **COMPETENT** | **SUPERIOR**  **PERFORMANCE** |
| --- | --- | --- | --- |

**COMMENTS:**

1. **WHAT WAS DONE WELL?**

**__________________________________________________________________________________________________________________________________________________________________________________________________________________**

1. **WHAT WAS NOT DONE WELL?**

**__________________________________________________________________________________________________________________________________________________________________________________________________________________**

1. **WHAT CAN BE IMPROVED?**

**__________________________________________________________________________________________________________________________________________________________________________________________________________________**

**SIGNED :___________________________________________**

**REPEAT ASSESSMENT**

**ASSESSED BY:_______________________________________________________**

**DATE:_____________________________________________________**

**ZONE OF PERFORMANCE (MBChB 3 level)**

| **FAILURE** | **WEAK PASS** | **COMPETENT** | **SUPERIOR**  **PERFORMANCE** |
| --- | --- | --- | --- |

**COMMENTS:**

1. **WHAT WAS DONE WELL?**

**______________________________________________________________________________________________________________________________________________________________________________________________________**

**2) WHAT WAS NOT DONE WELL?**

**______________________________________________________________________________________________________________________________________________________________________________________________________**

1. **WHAT CAN BE IMPROVED?**

**_________________________________________________________________**

**_________________________________________________________________**

**_________________________________________________________________**

**SIGNED:_______________________________________________________**

**PROCEDURAL SKILLS**

**A PRACTICAL APPROCH TO FUNDOSCOPY**

**ASSESSED BY:____________________________________________________________**

**DATE:____________________________________________________________________**

**ZONE OF PERFORMANCE (MBChB 3 level):**

| **FAILURE** | **WEAK PASS** | **COMPETENT** | **SUPERIOR**  **PERFORMANCE** |
| --- | --- | --- | --- |

**COMMENTS:**

**1) WHAT WAS DONE WELL?**

**_________________________________________________________________________**

**_________________________________________________________________________**

**2) WHAT WAS NOT DONE WELL?**

**___________________________________________________________________________________________________________________________________________________________________________________________________________________________**

**3) WHAT CAN BE IMPROVED?**

**____________________________________________________________________________________________________________________________________________________________________________________________________________________________________________________________________________________________________**

**SIGNED:___________________**

**A BASIC APPROACH TO X-RAYS OF SPINE, BONES AND JOINTS**

**ASSESSED BY:____________________________________________________________**

**DATE:____________________________________________________________________**

**ZONE OF PERFORMANCE (MBChB 3 level):**

| **FAILURE** | **WEAK PASS** | **COMPETENT** | **SUPERIOR**  **PERFORMANCE** |
| --- | --- | --- | --- |

**COMMENTS:**

**1) WHAT WAS DONE WELL?**

**______________________________________________________________**

**_________________________________________________________________________**

**2) WHAT WAS NOT DONE WELL?**

**___________________________________________________________________________________________________________________________________________________________________________________________________________________________**

**3) WHAT CAN BE IMPROVED?**

**____________________________________________________________________________________________________________________________________________________________________________________________________________________________________________________________________________________________________**

**SIGNED:___________________**

**THEME 3.3**

**ASSESSMENT OF CLINICAL EXAMINATION SKILLS**

**SKILL ASSESSED:__________________________________________________**

**ASSESSED BY:____________________________________________________________**

**DATE:____________________________________________________________________**

**ZONE OF PERFORMANCE (MBChB 3 level):**

| **FAILURE** | **WEAK PASS** | **COMPETENT** | **SUPERIOR**  **PERFORMANCE** |
| --- | --- | --- | --- |

**COMMENTS:**

**1) WHAT WAS DONE WELL?**

**______________________________________________________________**

**_________________________________________________________________________**

**2) WHAT WAS NOT DONE WELL?**

**___________________________________________________________________________________________________________________________________________________________________________________________________________________________**

**3) WHAT CAN BE IMPROVED?**

**____________________________________________________________________________________________________________________________________________________________________________________________________________________________________________________________________________________________________**

**SIGNED: ___________________**

**REPEAT ASSESSMENT**

**ASSESSED BY: ___________________________________________**

**DATE: ___________________________________________________**

**ZONE OF PERFORMANCE (**MBChB 3 level)

| **FAILURE** | **WEAK PASS** | **COMPETENT** | **SUPERIOR**  **PERFORMANCE** |
| --- | --- | --- | --- |

**COMMENTS:**

1. **WHAT WAS DONE WELL?**

**______________________________________________________________________________________________________________________________________________________________________________________________________**

1. **WHAT WAS NOT DONE WELL?**

**______________________________________________________________________________________________________________________________________________________________________________________________________**

1. **WHAT CAN BE IMPROVED?**

**______________________________________________________________________________________________________________________________________________________________________________________________________**

**SIGNED:___________________________________________________**

**PROCEDURAL SKILLS**

**GYNAECOLOGICAL EXAMINATION**

**ASSESSED BY: _________________________________________________________**

**DATE:____________________________________________________________________**

**ZONE OF PERFORMANCE (MBChB** 3 level)

| **FAILURE** | **WEAK PASS** | **COMPETENT** | **SUPERIOR**  **PERFORMANCE** |
| --- | --- | --- | --- |

**COMMENTS:**

1. **WHAT WAS DONE WELL?**

**_________________________________________________________________________________________________________________________________________________________________________________________________________**

1. **WHAT WAS NOT DONE WELL?**

**________________________________________________________________________________________________________________________________________________________________________________________________________**

1. **WHAT CAN BE IMPROVED?**

**_________________________________________________________________________________________________________________________________________________________________________________________________________**

**SIGNED:__________________________________________________________**

**OBSTETRIC ABDOMINAL EXAM**

**ASSESSED BY: ______________________________________________**

**DATE: __________________________________________________**

**ZONE OF PERFORMANCE (MBChB** 3 level)

| **FAILURE** | **WEAK PASS** | **COMPETENT** | **SUPERIOR**  **PERFORMANCE** |
| --- | --- | --- | --- |

**COMMENTS:**

1. **WHAT WAS DONE WELL?**

**_________________________________________________________________________________________________________________________________________________________________________________________________________**

1. **WHAT WAS NOT DONE WELL?**

**___________________________________________________________________________________________________________________________________________________________________________________________________________________________**

1. **WHAT CAN BE IMPROVED?**

**___________________________________________________________________________________________________________________________________________________________________________________________________________________________**

**SIGNED:________________________________________________________**

**PAP SMEAR**

**ASSESSED BY: ___________________________________________________**

**DATE: ________________________________________________________________**

ZONE OF PERFORMANCE (MBChB 3 level)

| **FAILURE** | **WEAK PASS** | **COMPETENT** | **SUPERIOR**  **PERFORMANCE** |
| --- | --- | --- | --- |

**COMMENTS:**

1. **WHAT WAS DONE WELL?**

**_________________________________________________________________________________________________________________________________________________________________________________________________________**

1. **WHAT WAS NOT DONE WELL?**

**_________________________________________________________________________________________________________________________________________________________________________________________________________**

1. **WHAT CAN BE IMPROVED?**

**_________________________________________________________________________________________________________________________________________________________________________________________________________**

**SIGNED:___________________________________________________________**

**PARTOGRAM & MECHANISM OF LABOUR AND DELIVERY (USING THE MODEL)**

**ASSESSED BY: _________________________________**

**DATE: ____________________________________________**

**ZONE OF PERFORMANCE** (MBChB 3 level)

| **FAILURE** | **WEAK PASS** | **COMPETENT** | **SUPERIOR**  **PERFORMANCE** |
| --- | --- | --- | --- |

**COMMENTS:**

1. **WHAT WAS DONE WELL?**

**__________________________________________________________________________________________________________________________________________________________________________________________________________________**

1. **WHAT WAS NOT DONE WELL?**

**__________________________________________________________________________________________________________________________________________________________________________________________________________________**

1. **WHAT CAN BE IMPROVED?**

**__________________________________________________________________________________________________________________________________________________________________________________________________________________**

**SIGNED;___________________________________**

**EXAMINATION OF THE MALE EXTERNAL GENITALIA**

**ASSESSED BY:____________________________________________**

**DATE:___________________________________________________**

**ZONE OF PERFORMANCE (**MBChB 3 level)

| **FAILURE** | **WEAK PASS** | **COMPETENT** | **SUPERIOR**  **PERFORMANCE** |
| --- | --- | --- | --- |

**COMMENTS:**

1. **WHAT WAS DONE WELL?**

**_________________________________________________________________________________________________________________________________________________________________________________________________________**

1. **WHAT WAS NOT DONE WELL?**

**_________________________________________________________________________________________________________________________________________________________________________________________________________**

1. **WHAT CAN BE IMPROVED?**

**________________________________________________________________________________________________________________________________________________________________________________________________________**

**SIGNED:__________________________________________________**

**RECTAL EXAMINATION**

**ASSESSED BY: ____________________________________________**

**DATE: ____________________________________________________**

**ZONE OF PERFORMANCE** (MBChB 3 level)

| **FAILURE** | **WEAK PASS** | **COMPETENT** | **SUPERIOR**  **PERFORMANCE** |
| --- | --- | --- | --- |

**COMMENTS:**

1. **WHAT WAS DONE WELL?**

**__________________________________________________________________________________________________________________________________________________________________________________________________________________**

1. **WHAT WAS NOT DONE WELL?**

**___________________________________________________________________________________________________________________________________________________________________________________________________________________________**

1. **WHAT CAN BE IMPROVED?**

**___________________________________________________________________________________________________________________________________________________________________________________________________________________________**

**SIGNED:____________________________________________**

**THEME 3.4**

**ASSESSMENT OF CLINICAL EXAMINATION SKILLS**

**SKILL ASSESSED:_____________________________________**

**ASSESSED BY:_____________________________________________**

**DATE:______________________________________________________**

**ZONE OF PERFORMANCE** (MBChB 3level)

| **FAILURE** | **WEAK PASS** | **COMPETENT** | **SUPERIOR**  **PERFORMANCE** |
| --- | --- | --- | --- |

**COMMENTS:**

1. **WHAT WAS DONE WELL?**

**__________________________________________________________________________________________________________________________________________________________________________________________________________________**

1. **WHAT WAS NOT DONE WELL?**

**__________________________________________________________________________________________________________________________________________________________________________________________________________________**

1. **WHAT CAN BE IMPROVED?**

**__________________________________________________________________________________________________________________________________________________________________________________________________________________**

**SIGNED:____________________________________________________**

**PROCEDURAL SKILLS**

**BEDSIDE HAEMOGLOBIN**

**ASSESSED BY:____________________________________________________________**

**DATE:____________________________________________________________________**

**ZONE OF PERFORMANCE (MBChB 3 level):**

| **FAILURE** | **WEAK PASS** | **COMPETENT** | **SUPERIOR**  **PERFORMANCE** |
| --- | --- | --- | --- |

**COMMENTS:**

**1) WHAT WAS DONE WELL?**

**_________________________________________________________________________**

**_________________________________________________________________________**

**2) WHAT WAS NOT DONE WELL?**

**___________________________________________________________________________________________________________________________________________________________________________________________________________________________**

**3) WHAT CAN BE IMPROVED?**

**____________________________________________________________________________________________________________________________________________________________________________________________________________________________________________________________________________________________________**

**SIGNED:__________________________________________________**

**THEME 3.5**

**ASSESSMENT OF CLINICAL EXAMINATION SKILLS**

**SKILL ASSESSED:__________________________________________________**

**ASSESSED BY:____________________________**

**DATE:___________________________________________**

**ZONE OF PERFORMANCE** ( MBChB3 level)

| **FAILURE** | **WEAK PASS** | **COMPETENT** | **SUPERIOR**  **PERFORMANCE** |
| --- | --- | --- | --- |

**COMMENTS:**

1. **WHAT WAS DONE WELL?**

**___________________________________________________________________________________________________________________________________________________________________________________________________________________________**

1. **WHAT WAS NOT DONE WELL? ___________________________________________________________________________________________________________________________________________________________________________________________________________________________**
2. **WHAT CAN BE IMPROVED?**

**___________________________________________________________________________________________________________________________________________________________________________________________________________________________**

**SIGNED:__________________________________________**

**REPEAT ASSESSMENT**

**ASSESSED BY:___________________________________**

**DATE:_____________________________________**

**ZONE OF PERFORMANCE** (MBChB 3 level)

| **FAILURE** | **WEAK PASS** | **COMPETENT** | **SUPERIOR**  **PERFORMANCE** |
| --- | --- | --- | --- |

**COMMENTS:**

1. **WHAT WAS DONE WELL?**

**_______________________________________________________________________________________________________________________________________________________________________________________________________________**

1. **WHAT WAS NOT DONE WELL?**

**________________________________________________________________________________________________________________________________________________________________________________________________________________________**

1. **WHAT CAN BE IMPROVED?**

**________________________________________________________________________________________________________________________________________________________________________________________________________________________**

**SIGNED:____________________________**

**PROCEDURAL SKILLS**

**RAPID HIV TESTING**

**ASSESSED BY:____________________________________________________________**

**DATE:____________________________________________________________________**

**ZONE OF PERFORMANCE (MBChB 3level):**

| **FAILURE** | **WEAK PASS** | **COMPETENT** | **SUPERIOR**  **PERFORMANCE** |
| --- | --- | --- | --- |

**COMMENTS:**

**1) WHAT WAS DONE WELL?**

**______________________________________________________________**

**_________________________________________________________________________**

1. **WHAT WAS NOT DONE WELL?**

**___________________________________________________________________________________________________________________________________________________________________________________________________________________________**

1. **WHAT CAN BE IMPROVED?**

**___________________________________________________________________________________________________________________________________________________________________________________________________________________________**

**SIGNED: _________________________________________________________________**

**TB TESTING IN CHILDREN: MANTOUX, SPUTUM COLLECTION, GASTRIC WASHING**

**ASSESSED BY:________________________________________________**

**DATE:______________________________________**

ZONE OF PERFORMANCE (MBChB 3 level)

| **FAILURE** | **WEAK PASS** | **COMPETENT** | **SUPERIOR**  **PERFORMANCE** |
| --- | --- | --- | --- |

**COMMENTS:**

1. **WHAT WAS DONE WELL?**

**__________________________________________________________________________________________________________________________________________________________________________________________________________________**

1. **WHAT WAS NOT DONE WELL?**

**__________________________________________________________________________________________________________________________________________________________________________________________________________________**

1. **WHAT CAN BE IMPROVED?**

**__________________________________________________________________________________________________________________________________________________________________________________________________________________**

**SIGNED:______________________________________________________________**

**URINE AND STOOLS SPECIMENS**

**ASSESSED BY:_______________________________________________________**

**DATE:___________________________________________**

**ZONE OF PERFORMANCE** ( MBChB 3 level)

| **FAILURE** | **WEAK PASS** | **COMPETENT** | **SUPERIOR**  **PERFORMANCE** |
| --- | --- | --- | --- |

**COMMENTS:**

1. **WHAT WAS DONE WELL?**

**_________________________________________________________________________________________________________________________________________________________________________________________________________**

1. **WHAT WAS NOT DONE WELL?**

**_________________________________________________________________________________________________________________________________________________________________________________________________________**

1. **WHAT CAN BE IMPROVED?**

**________________________________________________________________________________________________________________________________________________________________________________________________________**

**SIGNED:______________________________________________________**

**WOUND SPECIMENS, FUNGAL SCRAPE AND PUS ASPIRATE**

**ASSESSED BY:_____________________________________________**

**DATE:_________________________________________________**

**ZONE OF PERFORMANCE** ( MBChB 3 level)

| **FAILURE** | **WEAK PASS** | **COMPETENT** | **SUPERIOR**  **PERFORMANCE** |
| --- | --- | --- | --- |

**COMMENTS:**

1. **WHAT WAS DONE WELL?**

**_________________________________________________________________________________________________________________________________________________________________________________________________________**

1. **WHAT WAS NOT DONE WELL?**

**_________________________________________________________________________________________________________________________________________________________________________________________________________**

1. **WHAT CAN BE IMPROVED?**

**_________________________________________________________________________________________________________________________________________________________________________________________________________**

**SIGNED;_____________________________________________**

**GENITAL SPECIMENS (male and female)**

**ASSESSED BY:__________________________________**

**DATE:__________________________________________**

**ZONE OF PERFORMANCE** ( MBChB 3level)

| **FAILURE** | **WEAK PASS** | **COMPETENT** | **SUPERIOR**  **PERFORMANCE** |
| --- | --- | --- | --- |

**COMMENTS:**

1. **WHAT WAS DONE WELL?**

**_________________________________________________________________________________________________________________________________________________________________________________________________________**

1. **WHAT WAS NOT DONE WELL?**

**_________________________________________________________________________________________________________________________________________________________________________________________________________**

1. **WHAT CAN BE IMPROVED?**

**_________________________________________________________________________________________________________________________________________________________________________________________________________**

**SIGNED:___________________________________________**

**MEASUREMENT OF BODY TEMPERATURE**

**ASSESSED BY:__________________________________**

**DATE:__________________________________________**

ZONE OF PERFORMANCE ( MBChB 3level)

| **FAILURE** | **WEAK PASS** | **COMPETENT** | **SUPERIOR**  **PERFORMANCE** |
| --- | --- | --- | --- |

**COMMENTS:**

1. **WHAT WAS DONE WELL?**

**_________________________________________________________________________________________________________________________________________________________________________________________________________**

1. **WHAT WAS NOT DONE WELL?**

**_________________________________________________________________________________________________________________________________________________________________________________________________________**

1. **WHAT CAN BE IMPROVED?**

**_________________________________________________________________________________________________________________________________________________________________________________________________________**

**SIGNED:___________________________________________**

**BLOOD CULTURE & BLOOD COLLECTION USING THE VACUTAINER**

**ASSESSED BY:____________________________________________**

**DATE:________________________________________________________**

ZONE OF PERFORMANCE (MBChB 3 level)

| **FAILURE** | **WEAK PASS** | **COMPETENT** | **SUPERIOR**  **PERFORMANCE** |
| --- | --- | --- | --- |

**COMMENTS:**

1. **WHAT WAS DONE WELL?**

**_________________________________________________________________________________________________________________________________________________________________________________________________________**

1. **WHAT WAS NOT DONE WELL?**

**_________________________________________________________________________________________________________________________________________________________________________________________________________**

1. **WHAT CAN BE IMPROVED?**

**_________________________________________________________________________________________________________________________________________________________________________________________________________**

**SIGNED;_____________________________________________**

**NEONATAL RESUSCITATION**

**ASSESSED BY;_______________________________________**

**DATE:________________________________________________________**

**ZONE OF PERFORMANCE** ( MBChB 3 level)

| **FAILURE** | **WEAK PASS** | **COMPETENT** | **SUPERIOR**  **PERFORMANCE** |
| --- | --- | --- | --- |

**COMMENTS:**

1. **WHAT WAS DONE WELL?**

**__________________________________________________________________________________________________________________________________________________________________________________________________________________**

1. **WHAT WAS NOT DONE WELL?**

**__________________________________________________________________________________________________________________________________________________________________________________________________________________**

1. **WHAT CAN BE IMPROVED?**

**__________________________________________________________________________________________________________________________________________________________________________________________________________________**

**SIGNED:__________________________________**

**HAND HYGIENE, GLOVING & GOWNING**

**ASSESSED BY:____________________________________________**

**DATE:___________________________________________**

**ZONE OF PERFORMANCE** (MBChB 3 level)

| **FAILURE** | **WEAK PASS** | **COMPETENT** | **SUPERIOR**  **PERFORMANCE** |
| --- | --- | --- | --- |

**COMMENTS:**

1. **WHAT WAS DONE WELL?**

**_________________________________________________________________________________________________________________________________________________________________________________________________________**

1. **WHAT WAS NOT DONE WELL?**

**_________________________________________________________________________________________________________________________________________________________________________________________________________**

1. **WHAT CAN BE IMPROVED?**

**_________________________________________________________________________________________________________________________________________________________________________________________________________**

**SIGNED:________________________________________________________**
